# Novel polyethyleneimine-R8-heparin nanogel for high-efficiency gene delivery *in vitro* and *in vivo*

**DOI:** 10.1080/10717544.2017.1417512

**Published:** 2017-12-21

**Authors:** Linjiang Song, Xiuqi Liang, Suleixin Yang, Ning Wang, Tao He, Yan Wang, Lan Zhang, Qinjie Wu, Changyang Gong

**Affiliations:** ^a^ State Key Laboratory of Biotherapy and Cancer Center, West China Hospital, Sichuan University, and Collaborative Innovation Center for Biotherapy Chengdu P. R. China; ^b^ Personalized Drug Therapy Key Laboratory of Sichuan Province, Hospital of the University of Electronic Science and Technology of China and Sichuan Provincial People’s Hospital Chengdu P. R. China; ^c^ Research and Development Department, Guangdong Zhongsheng Pharcacy Dongguan China

**Keywords:** Nanogel, polyethyleneimine, R8, heparin, gene delivery

## Abstract

Gene therapy is an efficient and promising approach to treat malignant tumors. However, protecting the nucleic acid from degradation *in vivo* and efficient delivering it into tumor cells remain challenges that require to be addressed before gene therapy could be applied in clinic. In this study, we prepared novel polyethyleneimine-RRRRRRRR(R8)-heparin (HPR) nanogel as an efficient gene delivery system, which consists of heparin and cell penetrating peptide R8 grafted low-molecule-weight polyethyleneimine (PEI). Due to the shielding effect of heparin, crosslinking PEI-R8 with heparin was designed to diminish the toxicity of the gene delivery system. Meanwhile, a partial of R8 peptide which located on the surface of HPR nanogel could significantly enhance the cellular uptake. The formed HPR/pDNA complex exhibited effective endolysosomal escape, resulting in a high-efficiency transfection. Furthermore, the HPR could deliver the plasmid which could transcribe human TNF-related apoptosis inducing ligand (phTRAIL), into HCT-116 cells and induce significant cell apoptosis. In addition, HPR/phTRAIL complex showed satisfactory antitumor activity in abdominal metastatic colon carcinoma model. Finally, the antitumor mechanism of HPR/phTRAIL was also explored by western blot and histological analysis. The above results suggested that the HPR nanogel could serve as a promising gene delivery system.

## Introduction

Increasing attention has been focused on gene therapy, because it provides a promising approach to treat many diseases including cancers by delivering genetic drugs into target cells and tissues (Yang et al., 2007; Wang et al., 2012). Naked DNA is unsuitable for *in vivo* application, as it is easily degraded by serum nucleases and rapidly eliminated by renal excretion (Bumcrot et al., 2006; Kim et al., 2016). Therefore, a suitable gene delivery system that could protect nucleic acid from degradation and selectively deliver them into target cells is needed. Even though the viral vectors exhibited high transfection efficiency, it is limited to further application in clinic because of the inherent immunogenicity, insertional mutagenesis and possibly carcinogenicity (Lehrman, 1999; Kay et al., 2001). Recently, nonviral vectors have attracted more attention in gene therapy. Nonviral vectors, such as cationic polymers, dendrimers, lipids and peptides, are characterized by low immunogenicity and facile fabrication and thus used as promising gene delivery system (Elsabahy et al., 2011; Yin et al., 2014; Jeong et al., 2016).

Polyethyleneimine (PEI) is an important category of cationic polymer nonviral vector, which can compact the anionic nucleic acid into nanocomplex. The complex entered tumor cells through endocytotic pathway and endosomal escape effect and exhibited high transfection efficiency (Lungwitz et al., 2005). However, the molecular weight (MW) of the PEI influenced the balance between transfection efficiency and toxicity. For example, high MW (e.g. 25,000) PEI exhibits high transfection efficiency, but the toxicity is also high. On the contrary, low MW (e.g. 1800) PEI showed low transfection efficiency with low toxicity (Nakayama, 2012; Teo et al., 2013) The above limitations restricted PEI to further *in vivo* applications (Xu et al., 2009; Ganesh et al., 2013; Qi et al., 2016; Qin et al., 2016).

With the attempt to solve the above problems associated with PEI-mediated gene delivery, we prepared a high-efficiency transfection and low cytotoxicity PEI- RRRRRRRR(R8)-heparin (HPR) nanogel. We grafted a cell penetrating peptide R8 onto PEI 1.8 K (PEI-R8) to increase the charge density, and thus enhance cellular uptake and gene transfection efficiency (Yin et al., 2013; Douat et al., 2015). Heparin is a natural anionic polysaccharide consisted of highly sulfated repeating units. It is nontoxic, biodegradable and shows many special properties such as anticoagulation of blood and inhibition of angiogenesis in physiology (Lv et al., 2015). Therefore, we used heparin as the skeleton of our gene delivery system to diminish the toxicity of PEI. PEI-R8 was then conjugated with heparin to form the HPR nanogel gene delivery system. Cellular uptake is essential in gene delivery, and the positive charges of PEI and R8 in the nanogel could enhance cellular uptake through charge-mediated interactions with negative charges presented on the cell surface (Mislick & Baldeschwieler, 1996; Vercauteren et al., 2011; Dong et al., 2015). In addition, a partial of the tumor cell penetrating peptide R8 which located on the surface of nanogel also enhanced endocytosis-uptake of the nanogel (Nomura et al., 2015; Tang et al., 2017). After cellular uptake, the endolysosomal escape which resulted from the proton-sponge effect of PEI-R8 promote the nanogel enter into the nuclei of cells (Li et al., 2016a).

In the current study, we synthesized novel HPR nanogel and evaluated the transfection efficiency and endolysosomal escape of HPR/plasmid DNA (pDNA) complex *in vitro*. Meanwhile, we employed the HPR nanogel to deliver human TNF-related apoptosis inducing ligand plasmid (phTRAIL), a pDNA which could encode human TNF-related apoptosis inducing ligand (hTRAIL), into tumor cells to induce cancer cell apoptosis. Moreover, we evaluated the therapeutic efficiency of HPR/phTRAIL complex in abdominal metastatic colon carcinoma model *in vivo*.

## Materials and methods

### Materials, cells and animals

Branched PEI with MW of 1.8 KDa and 25 KDa, heparin, 1-(3-dimethylaminopropyl)-3-ethylcarbodiimide (EDCI), N-Hydroxysuccinimide (NHS), 4-morpholineethanesulfonic acid (MES), 3-(4,5-dimethylthiazol-2-yl)-2,5-diphenyl tetrazolium bromide (MTT) and Dulbecco’s modified Eagle’s medium (DMEM) were purchased from SigmaAldrich (USA). R8 (cys-Arg-Arg-Arg-Arg-Arg-Arg-Arg-Arg) was obtained from ChinaPeptides Co., Ltd (Shanghai China). N-Succinimidyl 3-maleimidopropionate was purchased from Research Accelerators Technology Co., Ltd. (Chengdu China). YOYO-1, Lyso-Tracker Red and DAPI were provided by Invitrogen (USA). Annexin V-FITC/PI apoptosis detection kit was purchased from KeyGen Biotech. Co., Ltd (Nanjing, China). The phTRAIL, empty vector plasmid control (pMCS) and green fluorescent protein plasmid (pGFP) were obtained from Invitrogen (USA), and amplified in DH-5a *Escherichia coli* and purified with the Qiagen PlasmidMega Kit (Qiagen, CA, USA).

HCT-116 cells which purchased from American Type Culture Collection (Rockville, Maryland) were cultured in DMEM medium (Gibco, Invitrogen, Grand Island, NY, USA) with 10% fetal bovine serum (FBS) and antibiotics at 37 °C with a humidified 5% CO_2_ atmosphere.

BALB/c nude mice (6–8 weeks) were provided by HFK Bio-Technology Company (Beijing, China). All the animals were sex-separately housed in specific pathogen-free (SPF) conditions and would be in quarantine for a week before treatment. All animal procedures were performed following the protocol approved by the Institutional Animal Care and Treatment Committee of Sichuan University (Chengdu, P.R. China). All animals were treated humanely throughout the experimental period.

### Preparation of HPR nanogel

PEI-R8 was synthesized as follows: PEI 1.8 K (1 g, 0.55 mmol, 1.0eq) was dissolved in 10 mL chloroform in a 25-mL round flask and then N-Succinimidyl 3-maleimidopropionate (177 mg, 0.666 mmol, 1.2eq) was added to the above solution. After stirring the mixture at room temperature for 3 h, the solvent was removed by rotary evaporation. The residues were dissolved with 10 mL THF/H_2_O (1:1, v/v) and reacted with cys-R8 (760 mg, 0.55 mmol, 1.0 eq) at room temperature for 6 hours. When the reaction was completed, the mixture was dialyzed (MWCO: 1000) against doubled distilled water to removed THF and unreacted N-succinimidyl 3-maleimidopropionate. The obtained products were lyophilized and stored in −20 °C for further use.

HPR nanogel was prepared through the amide linkage. In detail, 50 mg of heparin was dissolved in 100 mL of MES buffer (PH 5.5, 0.05 M), followed by adding into 30 mg of EDCI and 30 mg of NHS to activated the carboxylic acid groups of heparin. After stirring the mixture at room temperature for 3 h, the activated heparin solution was dropped into a consistently stirred 20 mL of PEI 1.8 K solution, which consisted of 30% of PEI-R8. When the mixture was stirred at room temperature for 12 h, the solution was dialyzed (MWCO: 10,000) against doubled distilled water for 3 days. The water was changed three times per day during the dialysis period. After filtering the HPR nanogel with a syringe filter (0.22 μm, Millipore Co., Billerica, MA, USA), the concentration of the HPR nanogel were adjusted to 1 mg/mL.

### Characterization of HPR nanogel and HPR/pDNA complex

The HPR/pDNA complex was formed through an electronic interaction. Briefly, 40 μg of HPR nanogel and 2 μg of phTRAIL plasmid solution were mixed gently, and then, the mixed solutions were incubated at room temperature for 25 min to form steady HPR/pDNA complex. The particle size and surface charges of the HPR and HPR/pDNA were measured by dynamic light scattering (DLS) measurement (Malvern, Zetasizer NanoZS ZEN 3600, UK). The morphologies of the nanogel and complex were observed by TEM (JEOL JEM;100CX, Japan).

The gel retardation experiments were carried as follows. HPR/phTRAIL complex was prepared at various mass ratio (1:10, 1:5, 1:1, 5:1, 10:1, 15:1, 20:1, HPR: phTRAIL), and the quantity of plasmid was constant. Then, HPR/phTRAIL complexes were electrophoresed on 1% (w/v) agarose gel at 120 V for 30 min. The gel was stained with GelView (BioTeke Co., Ltd, China). The DNA retardation was visualized by a UV illuminator using a Gel Doc System (Bio-Rad).

### *In vitro* cytotoxicity assessment

The toxicity of the PEI 25 K, PEI 1.8 K and HPR nanogel were evaluated by MTT assay. HCT-116 and HEK-293 cells were seeded in 96-well plates at a density of 5000 cells/well and then incubated for 12 h. The cells were treated with PEI 25 K, PEI 1.8 K and HPR at different concentrations (5, 10, 20, 40, 60, 80, and 120 μg/mL) for 48 h. After that, 20 μL of MTT (5 mg/mL) was added into the wells and the cells were incubated for 4 h at 37 °C. Finally, 150 μL of DMSO was added into each well to dissolve the precipitated formazan, and the absorbance at 570 nm was measured. All experiments were performed in triplicate. Data are expressed as means ± standard deviations (SD).

### Endolysosomal escape

phTRAIL was labeled with YOYO-1 according to the manufacturer’s protocol. HCT-116 cells were seeded in 6-well plate over glass cover slips at a density of 2 × 10^5^ cells/well and incubated for 12 h. The HPR nanogel loaded with 2 μg of YOYO-1 labeled phTRAIL were added into each well. At determined time (0.5, 1, 2, 4 h), HPR/phTRAIL-YOYO-1 complex was removed and cells were washed with PBS (PH 7.4). Then, lysosomes were labeled with Lyso-Tracker Red, and nuclei were stained with DAPI. Subsequently, cells were washed with PBS again and fixed with 4% paraformaldehyde. Finally, the cells were observed with Laser Scanning Confocal Microscope (LSCM) (Leica, TCS SP5, Germany).

### *In vitro* transfection study

Plasmid encoding GFP (pGFP) was used as a reporter gene to evaluate the gene transfection efficiency of the HPR nanogel. HCT-116 cells were seeded in 6-well plates at a density of 2 × 10^5^ cells/well and incubated for 12 h. Then, the culture medium was replaced with 1 mL DMEM containing no serum and no antibiotic. The HPR nanogel, PEI 1.8 K and PEI 25 K which all loaded with 2 μg of pGFP were added to the corresponding wells. After incubation for 6 h, the medium was replaced with 2 mL fresh completed DMEM medium and cells were incubated at 37 °C for another 24 h. Finally, expression efficiency of GFP was observed through a fluorescence microscope (Olympus, Japan) and quantified the transfection efficiency by flow cytometry (Calibur, BD, USA). All the transfection experiments were carried in triplicate.

### *In vitro* apoptosis evaluation

HCT-116 cells were seeded in 6-well plates and transfected with HPR nanogel which loaded with 1 μg of phTRAIL. Twenty-four hours later, cells were washed, collected and processed by Annexin V-FITC/PI apoptosis detection kit according to the protocol. Afterwards, the cells were analyzed by the flow cytometry (Calibur, BD). In addition, we exploited western blot to detect the apoptosis-associated protein expressed by the cells after transfection. The cells were collected, washed, lysised and centrifuged. Concentration of total protein in the supernatant was determined by BCA protein assay kits. After the protein was separated by SDS-PAGE gel and transferred to the PVDF membranes, the membranes were blocked with nonfat milk. Then, the PVDF membranes were treated with antibodies against cleaved-caspase 3 and cleaved-caspase 9 at 4 °C overnight, respectively. Afterwards, the membranes were washed with TBST several times to remove the free antibodies, followed by incubation with HRP-conjugated corresponding antibodies at 37 °C for 50 min. Finally, the PVDF membranes were washed and imaged using the chemiluminescence (ECL) detection system.

### Anticancer activity of HPR/phTRAIL complex *in vivo*

Thirty female BALB/c nude mice were abdominal injection of HCT-116 cells (1 × 10^7^ cells per mice) to establish abdominal cavity metastatic model. Eight days later, mice were divided into 5 groups and intraperitoneally administered with normal saline (NS, 200 μL), HPR nanogel (100 μg), PEI 25 K/phTRAIL complex (PEI 25 K:5 μg, phTRAIL:5 μg), HPR/phTRAIL (HPR:100 μg, phTRAIL:5 μg) and HPR/pMCS (HPR:100 μg, pMCS:5 μg) once every three days, respectively. The treatment was stopped when the mice in the NS was moribund on the 15th day. Mice were sacrificed by cervical vertebra dislocation, and the blood samples were collected from orbit for blood chemistry profile analysis and complete blood count test. Tumors were harvested, weighted and numbered the nodules. After that, the tumors and the collected organs (heart, liver, spleen, lung and kidney) were fixed in 4% paraformaldehyde for further hematoxylin and eosin (H&E) staining and immunological histological chemistry (IHC) analysis. The tumor tissue apoptosis was carried by the TUNEL method. The TUNEL assay procedure was in accordance with the In Situ Cell Death Detection Kit (KeyGEN BioTECH, China).

### Statistical analysis

The statistical analysis was measured by using SPSS 17.0 software (Chicago, IL). The significance among the samples was evaluated using one-way ANOVA. Significant differences between groups were indicated by *p < .05, **p < .01 and ***p < .001, respectively.

## Results and discussion

### Preparation and characterization of HPR nanogel and HPR/pDNA complex

**Figure 1.**
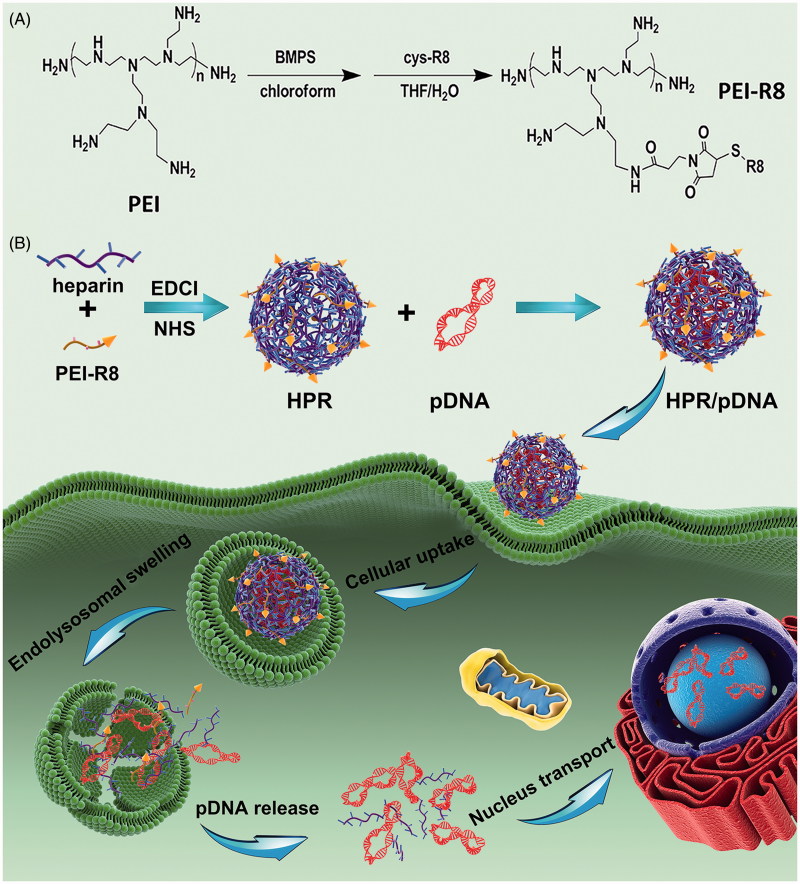
**(**A) Synthesized scheme of PEI-R8. (B) Schematic representation of the formation and *in vivo* gene therapy of HPR/phTRAIL nanoparticles.

For developing a high-efficiency and low toxic gene delivery system, we prepared HPR nanogel. PEI-R8 was synthesized by utilizing a linker which contains NHS ester and maleimide group to connect amine groups of PEI and thiol groups of R8 peptide. Then, reacting the carboxylic groups of heparin which activated by EDCI/NHS system with amine groups of PEI-R8 to form a HPR nanogel. The synthetic scheme is displayed in Figure 1. PEI not only reacted with heparin molecule but also acted as a linker which joint with two or more heparin molecules, resulting in a cross-linked nanogel.

**Figure 2.**
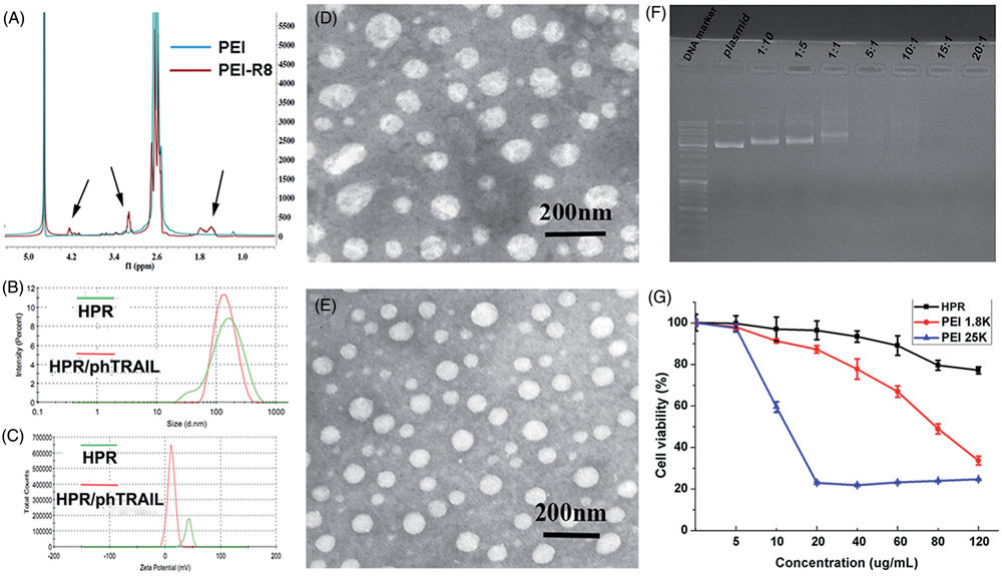
Characterization of HPR. (A) ^1 ^H-NMR spectrums of PEI and PEI-R8. (B) Size distribution of HPR and HPR/phTRAIL. (C) Zeta potential of HPR and HPR/phTRAIL. (D and E) TEM images of HPR and HPR/phTRAIL respectively. (F) Plasmid DNA condensation of HPR. (G) Cytotoxicity of HPR in HCT-116 cells.

PEI and PEI-R8 were characterized by ^1 ^H-NMR (Figure 2(A)). Compared with the PEI, new signals at 1.5 ppm, 2.8 ppm and 4.2 ppm were observed in the spectrum of PEI-R8, which correspond to the R8 peptide. The HPR nanogel displayed a diameter of 125 ± 3 nm and a positive zeta potential of +28 ± 2 mV (Figure 2(B,C)) which possessed ability to load a negative charge DNA. After that, we prepared the HPR/pDNA complex through an electronic interaction. Owing to the electronic interaction effect, HPR/pDNA turned into a more compacted nanosized complex in comparison with HPR nanogel. The HPR/pDNA complex showed a smaller diameter of 113 ± 2 nm and a positive zeta potential of +20 ± 3 mV (Figure 2(B,C)). Furthermore, we characterized the morphology of the HPR nanogel and HPR/pDNA complex by transmission electron microscopy (TEM), and both the nanogel and the complex displayed compact and spheroid morphology (Figure 2(D,E)).

To evaluate the ability of loading pDNA by HPR nanogel, we employed the gel retardation experiment. As shown in Figure 2(F), the HPR nanogel could not package the pDNA completely when the mass ratio of HPR/pDNA was lower than 1:1. On the contrary, when the mass ratio was 5:1 or higher, the pDNA band was disappeared, which indicated the formation of a compacted HPR/pDNA complex. Therefore, HPR nanogel showed well ability to efficiently load pDNA.

### Cytotoxicity studies

The cytotoxicity of HPR nanogel, PEI 1.8 K and PEI 25 K were investigated in HCT-116 cells by MTT assay. As displayed in Figure 2(G) and Figure S1, PEI 25 K and PEI1.8 K showed a dose-dependent cytotoxicity. Recent studies revealed that the cytotoxicity of cationic materials might result from the impairment of Na^+^/K^+^-ATPase (Wei et al., 2015). Inspired by strategies of shielding primary amines and neutralizing positive charges, we combined heparin with PEI-R8 and obtained a gene delivery system with low cytotoxicity. When connecting PEI1.8 K with heparin to form a nanogel, the cytotoxicity in HCT-116 and HEK-293 cells were decreased significantly compared with PEI1.8 K and PEI25K. The primary amine groups which could disrupt the protein kinase C were shielded by the grafted heparin (Yao et al., 2010). Meanwhile, the high positive charges of the PEI which were harm to cells could be partially neutralized by heparin (Nimesh et al., 2006; Jiang et al., 2007).

### Endolysosomal escape

**Figure 3.**
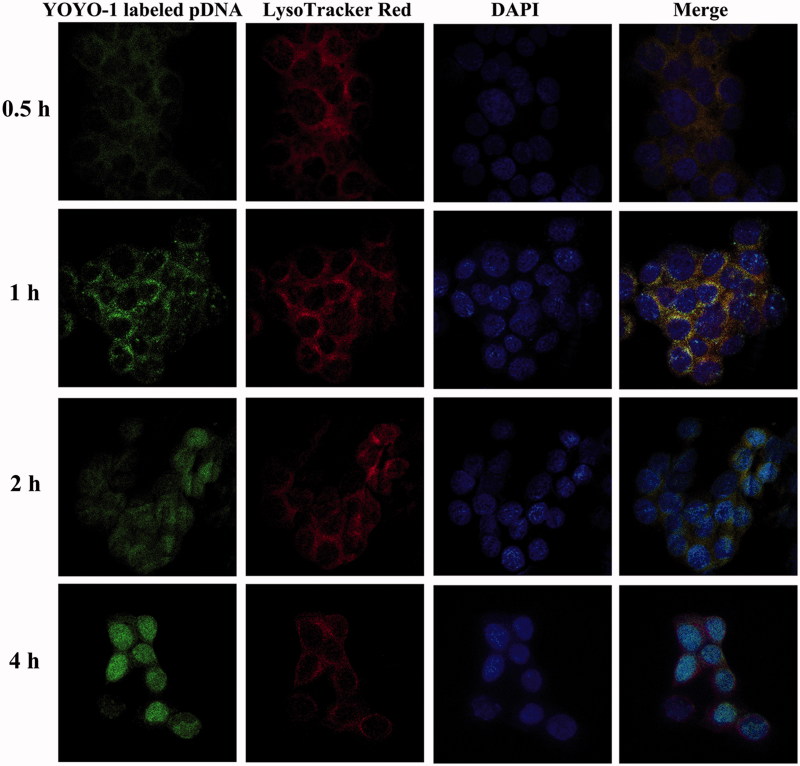
LSCM images of HCT-116 cells following incubation with HPR/pDNA nanoparticles at 37 °C for 0.5, 1, 2, 4 h. Plasmid DNA was labeled with YOYO-1. Endolysosomes were stained with LysoTracker-Red and cell nuclei were stained with DAPI.

For avoiding degradation by the enzymes in lysosomes, the encapsulated DNA should be protected by the delivery system that owns the ability of efficient endolysosomal escape (Yan et al., 2015; Yuan et al., 2015). Here, we evaluate the endolysosomal escape capacity of our HPR nanogel. As shown in Figure 3, HPR/phTRAIL complex was observed at the margin of the cell at the first 0.5 h, then entered cell and colocalized with lysosome within the first 2 h. Subsequently, the HPR/phTRAIL complex escaped from lysosome and penetrated into the nuclei of HCT-116 cells after 4 h incubation. Due to the PEI segment in the nanogel, HPR/pDNA complex showed ‘proton sponge effect’ to swell endolysosomal compartments, and then escaped from the endolysosomes and finally released pDNA into nuclei (Kircheis et al., 1997).

### *In vitro* gene transfection

**Figure 4.**
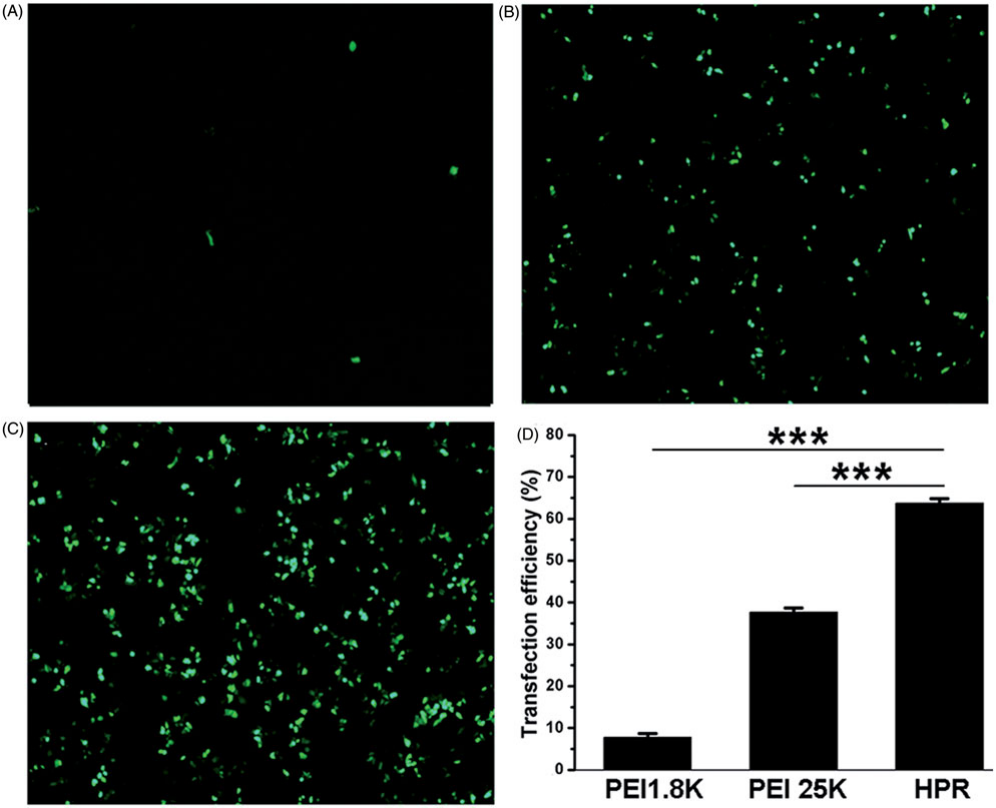
*In vitro* transfection efficiency of HPR. (A, B and C) Fluorescence microscopy images of HCT-116 cells after transfected with PEI1.8 K/pGFP, PEI25K/pGFP and HPR/pGFP respectively. (D) Quantitative analysis of transfection efficiency of PEI1.8 K, PEI25K and HPR.

*In vitro* gene transfection assay was performed using HCT-116 cells with various mass ratio of HPR/pGFP complex, and PEI25K was used as control. From our preliminary experiments, we found that the transfection efficiency was improved with an increase of mass ratio of HPR nanogel to pGFP. However, there was no obvious enhancement of transfection efficiency but accompany with severe cytotoxicity when the mass ratio was higher than 20:1 (data not shown). In consequence, we chose the mass ratio (20:1) of HPR to pGFP in our subsequent experiments. As presented in Figure 4, HPR/pGFP (20:1, mass ratio) complex displayed higher transfection efficiency (more than 64.3 ± 1.5%) at 24 h than that of PEI25K/pGFP (1:1, mass ratio) (less than 37.8 ± 1.6%). PEI1.8 K/pGFP showed low transfection efficiency in HCT-116 cells. The high gene transfection efficiency may result from the positive charges of the HPR/pDNA complex which promote interaction with the negative charges of cell membrane, as well as the cell-penetrating effect of R8 peptide located on the surface of HPR/pDNA complex.

### *In vitro* apoptosis

**Figure 5.**
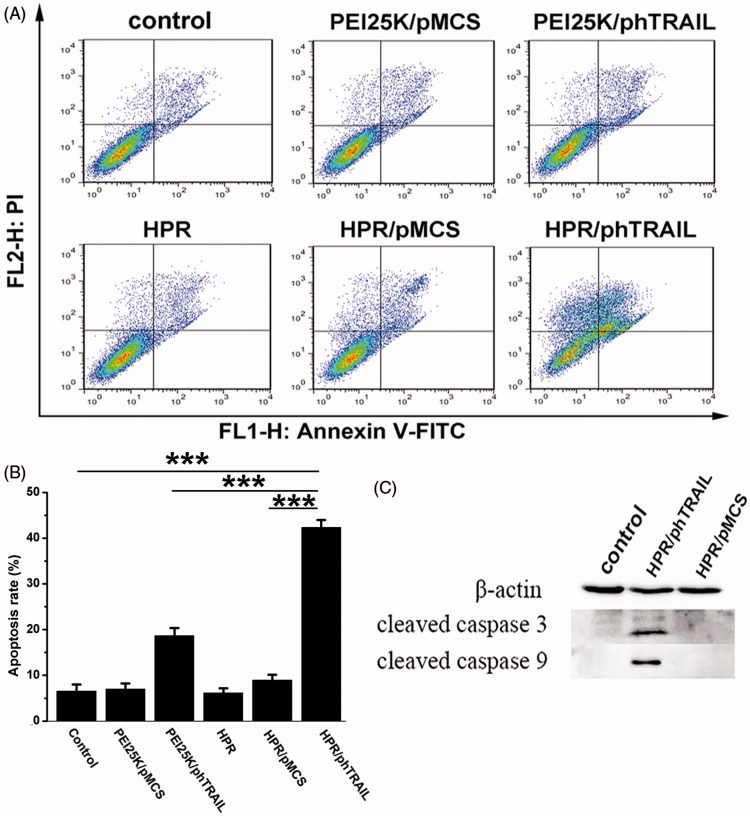
*In vitro* apoptosis evaluation. (A) Different treatments (NS, PEI25K/pMCS, PEI25K/phTRAIL, HPR, HPR/pMCS and HPR/phTRAIL) induced apoptosis in HCT-116 cells. Annexin V and PI-stained cells were measured by flow cytometry. (B) The percentages of Annexin V-positive HCT-116 cells after different treatments. (C) Expression levels of apoptosis-related proteins in HCT-116 cells detected by Western blot assay after different treatments.

hTRAIL has been identified as an apoptosis protein which could dramatically induce apoptosis of tumor cells without involving normal cells (Johnstone et al., 2002, 2008; Li et al., 2016b; Tao et al., 2017). In this study, phTRAIL, a plasmid which could express hTRAIL, was loaded by HPR nanogel and delivered into HCT-116 cells to induce apoptosis of tumor cells. As shown in Figure 5(A,B), HPR/phTRAIL complex treatment induced about 42.5 ± 1.8% of the total apoptotic ratio to HCT-116 cells, which was significantly higher than that of PEI25K/phTRAIL (18.9 ± 2.1% of the total apoptosis). HPR/pMCS rarely induced apoptosis (7.9 ± 1.6%). Cleaved-caspase 3 and cleaved-caspase 9 are key proteins which participate in the apoptosis signal of TRAIL (Wiley et al., 1995; Pitti et al., 1996). Figure 5(C) showed that the level of cleaved-caspase 3 and cleaved-caspase 9 were both enhanced in HPR/phTRAIL group. HPR nanogel can effectively deliver phTRAIL into HCT-116 cells to express hTRAIL protein, and then significantly induce cell apoptosis.

### Evaluation of antitumor efficiency *in vivo*

**Figure 6.**
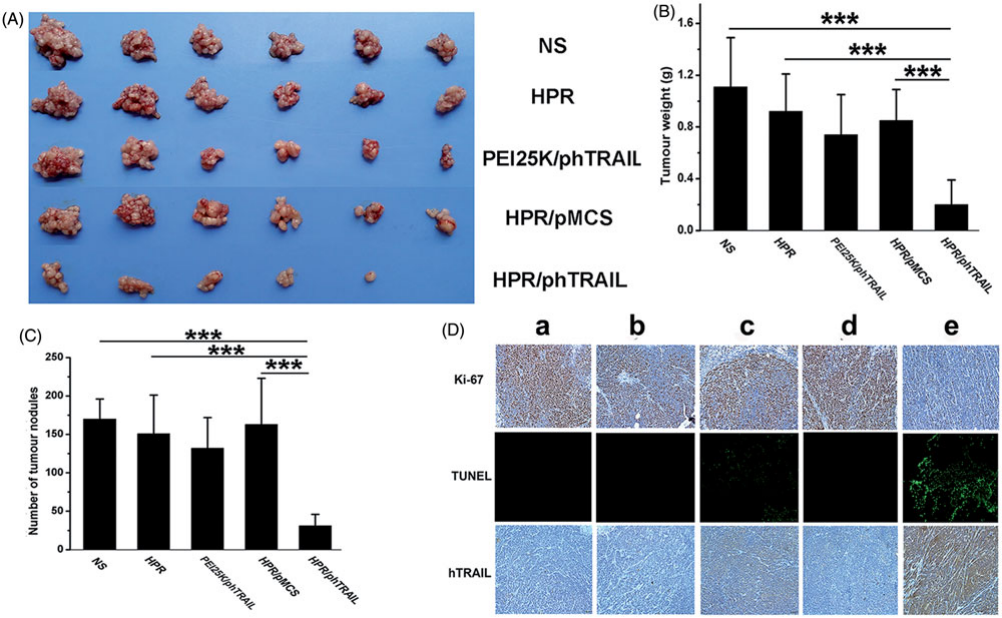
Antitumor effect of HPR/phTRAIL *in vivo*. (A) Photos of HCT-116 abdominal metastasis tumor after different treatments. (B and C) Tumor weight and nodules in different treatments. (D) Immunohistochemical analysis of Ki-67, TUNEL and hTRAIL expression of tumors in each group. (a) NS; (b) HPR; (c) PEI25K/phTRAIL; (d) HPR/pMCS; (e) HPR/phTRAIL.

On account of the good performance of HPR/phTRAIL *in vitro*, we evaluated the anticancer activity of HPR/phTRAIL in the abdominal cavity metastases model of HCT-116 colon carcinoma. As presented in Figure 6(A), HPR/phTRAIL significantly inhibited the tumor growth compared with other groups. The weight and nodules of the tumors were displayed in Figure 6(B,C). Compared with HPR/pMCS, HPR/phTRAIL caused a significant reduction in tumor weight and nodules, suggesting that the improved antitumor efficacy was resulted from the hTRAIL. HPR/phTRAIL showed a better antitumor activity than PEI25K/phTRAIL, implying that HPR nanogel is a more efficient gene delivery system than PEI 25 K.

### Histological analysis

Ki-67 is widely utilized as a proliferation maker in tumor prognosis (Whitfield et al., 2006; Yerushalmi et al., 2010). From Figure 6(D), compared with other treatment groups, an obvious decrease of Ki-67-positive tumor cells were found within the treatment of HPR/phTRAIL. Meanwhile, the TUNEL assay was carried to detect DNA fragmentation which resulted from cell apoptosis *in situ* (Heatwole, 1999). More cell apoptosis was observed in the HPR/phTRAIL treated tumor tissue, while the other four groups showed few apoptotic cells. To demonstrate that the results of TUNEL assay and Ki-67 were caused by apoptosis which resulted from the hTRAIL, we employed an immunohistochemical analysis to detect the expression of hTRAIL in tumors. As shown in Figure 6(D), consistent with the results of Ki-67 and TUNEL assay, high levels of hTRAIL were found in the HPR/phTRAIL group.

### Safety evaluation

Blood chemistry profile analysis, complete blood count (CBC) and histological analysis of major organs (heart, liver, spleen, lung and kidney) were performed for safety assessment (Figure S2 and Figure S3). The results of blood chemistry profile analysis and complete blood count among all groups were in normal range. Meanwhile, from the Figure S3, no obvious hematologic changes were observed in H&E staining of major organs in all treatment groups. Therefore, HPR nanogel could serve as a potential safe candidate as gene delivery system.

## Conclusions

We designed a novel HPR nanogel gene delivery system with enhanced transfection efficiency and low toxicity by cross-linking heparin with R8-grafted low-molecule-weight PEI. HPR nanogel could condense pDNA compactly and exhibit efficiently endolysosomal escape. Meanwhile, HPR nanogel presented high transfection efficiency and could efficiently deliver plasmid hTRAIL into HCT-116 cells to induce a significant apoptosis *in vitro*. Moreover, the HPR/phTRAIL complex exhibited prominent inhibition of tumor growth in mouse tumor model. Thus, the encouraging *in vitro* and *in vivo* performance suggests that HPR nanogel may serve as a potential gene delivery system in tumor therapy.

## Supplementary Material

IDRD_Gong_et_al_Supplemental_Content.doc
