# Correction

**DOI:** 10.1080/10717544.2019.1678695

**Published:** 2019-11-26

**Article title:** Novel polyethyleneimine-R8-heparin nanogel for high-efficiency gene delivery *in vitro* and *in vivo*.

**Authors:** Song, L., Liang, X., Yang, S., Wang, N., He, T., Wang, Y., Zhang, L., Wu, Q., & Gong, C.

**Journal:** *Drug Delivery*

**Bibliometrics:** Volume 25, Number 1, pages 122–131

**DOI:** https://doi.org/10.1080/10717544.2017.1417512.

When the above article was first published online, Figures 2(E) and 6(D) were incorrect. These have now been corrected. These mistakes do not influence any of the experimental results and discussion as well as conclusions reported in the paper.

**Figure 2.**
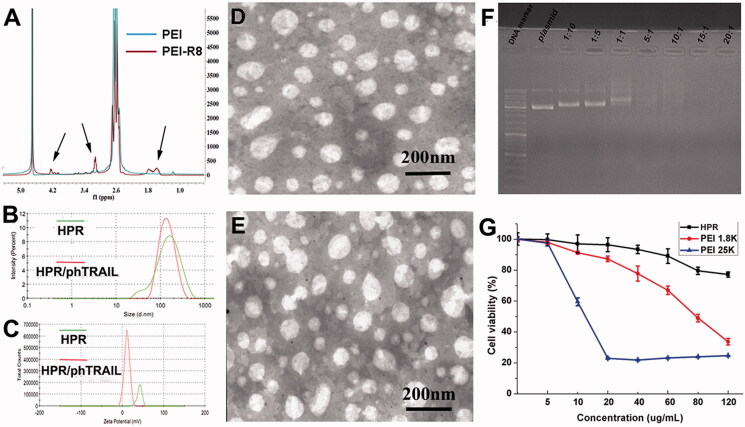
Characterization of HPR. A, ^1 ^H-NMR spectrums of PEI and PEI-R8. B, Size distribution of HPR and HPR/phTRAIL. C, Zeta potential of HPR and HPR/phTRAIL. D and E, TEM images of HPR and HPR/phTRAIL respectively. F, Plasmid DNA condensation of HPR. G, Cytotoxicity of HPR in HCT-116 cells.

**Figure 6.**
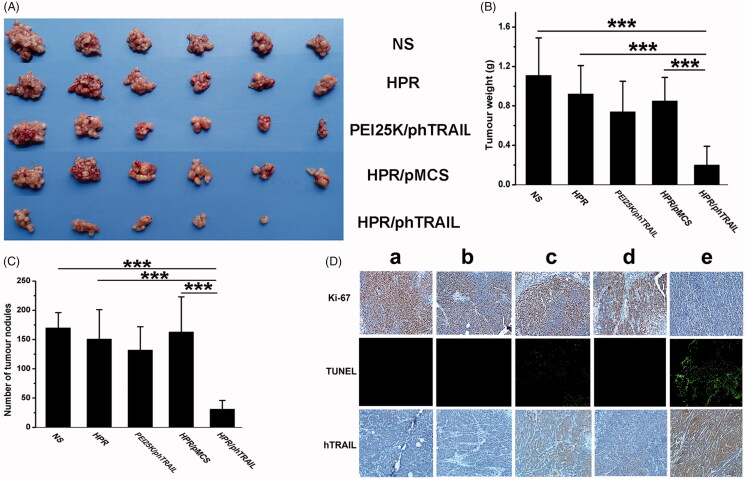
Antitumor effect of HPR/phTRAIL *in vivo*. A, Photos of HCT-116 abdominal metastasis tumor after different treatments. B and C Tumor weight and nodules in different treatments. D, Immunohistochemical analysis of Ki-67, TUNEL and hTRAIL expression of tumors in each group. a, NS; b, HPR; c, PEI25K/phTRAIL; d, HPR/pMCS; e, HPR/phTRAIL.

