# Supplementary material for: Novel polyethyleneimine-R8-heparin nanogel for high-efficiency gene delivery in vitro and in vivo
Source: Drug Deliv. 2017 Dec 21;25(1):122–31. doi: 10.1080/10717544.2017.1417512 (PMC6058572; doi:10.1080/10717544.2017.1417512)
Supplement: IDRD_Gong_et_al_Supplemental_Content.doc [file IDRD_A_1417512_SM8904.doc]

**Novel Polyethyleneimine-R8-heparin Nanogel for High-efficiency Gene Delivery *in vitro* and *in vivo***

Linjiang Song ^a,+^, Xiuqi Liang ^a,+^, Suleixin Yang ^a^, Ning Wang ^a^, Tao He ^a^, Yan Wang ^b^, Lan Zhang ^c^, Qinjie Wu ^a^, Changyang Gong ^a,*^

^a^ State Key Laboratory of Biotherapy and Cancer Center, West China Hospital, Sichuan University, and Collaborative Innovation Center for Biotherapy, Chengdu, 610041, P. R. China

^b^ Personalized Drug Therapy Key Laboratory of Sichuan Province, Hospital of the University of Electronic Science and Technology of China and Sichuan Provincial People’s Hospital, Chengdu, 610041, P. R. China

^c^ Research and Development Department, Guangdong Zhongsheng Pharcacy, Dongguan, 523325, China

^*^ To whom should be corresponded (C Gong). E-mail: [chygong14@163.com](mailto:chygong14@163.com).

^+^ These authors contributed equally to this work.

**
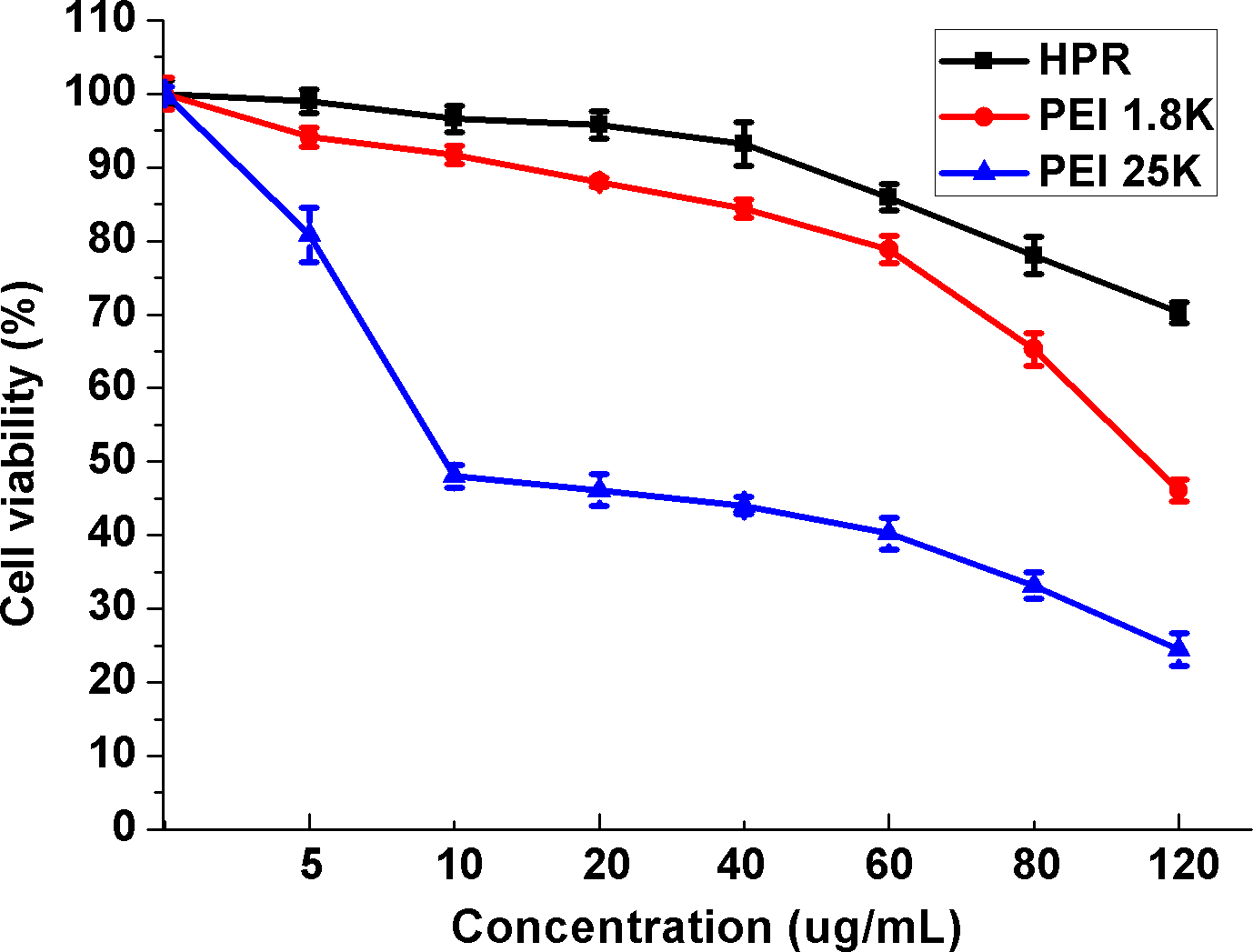
**

**Figure S1** Cytotoxicity of HPR, PEI 1.8K and PEI 25K in HEK-293 cells.


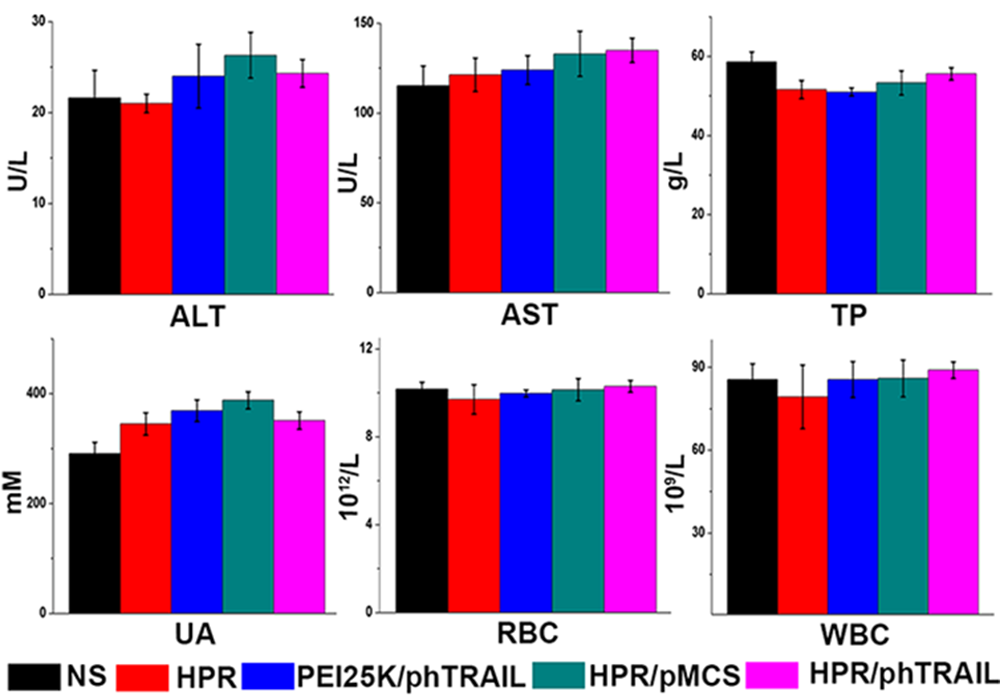


**Figure S2** Blood chemistry profile analysis and CBC test after different treatments.


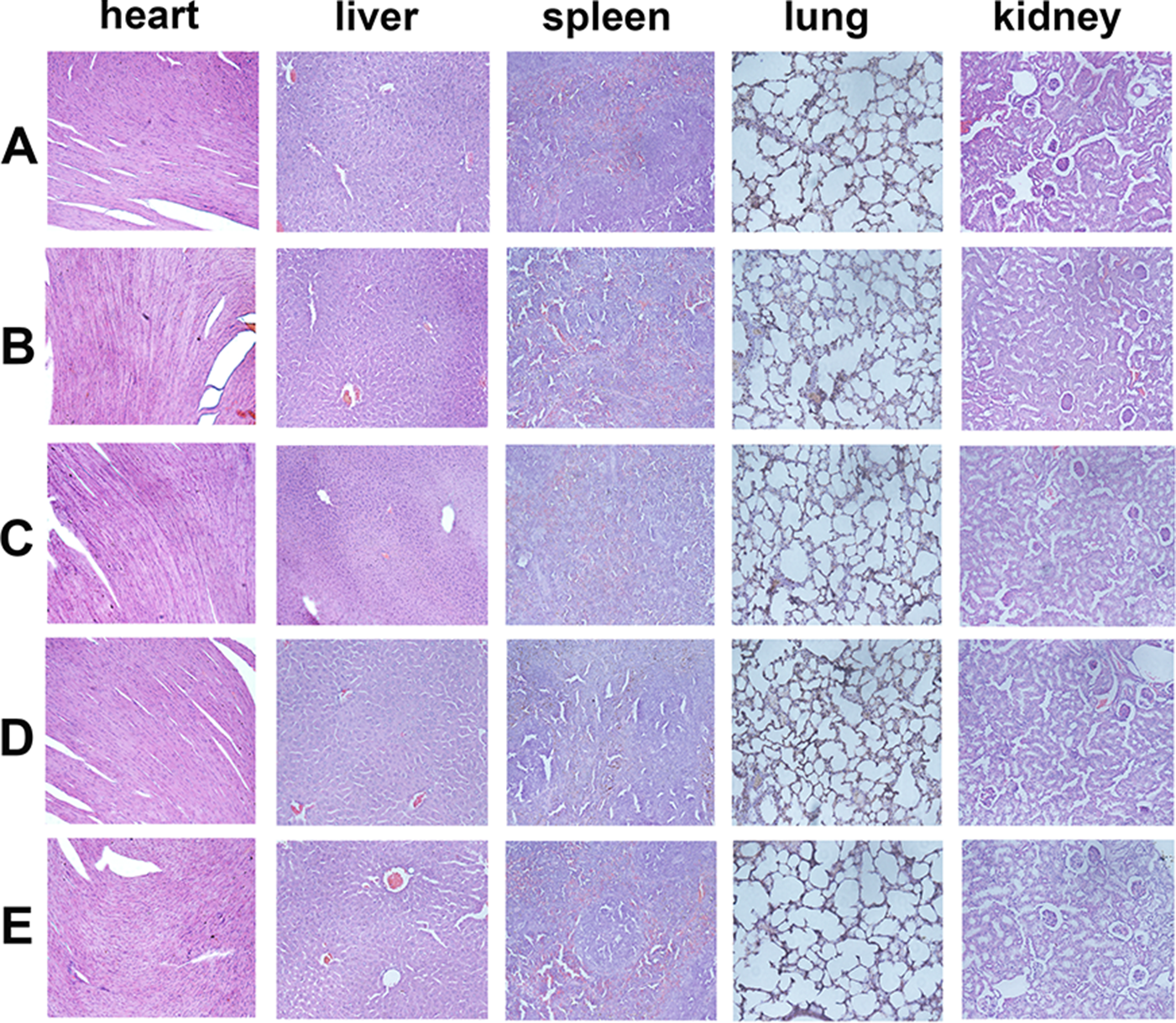


**Figure S3** Histological examination of H&E staining of organ sections. A, NS. B, HPR. C, PEI25K/phTRAIL. D, HPR/pMCS. E, HPR/phTRAIL.
